# Prevalence and Predictors of Combined Body Mass Index and Waist Circumference Among Indian Adults

**DOI:** 10.3389/ijph.2023.1605595

**Published:** 2023-03-29

**Authors:** Neha Shri, Saurabh Singh, Akancha Singh

**Affiliations:** ^1^ Department of Survey Research and Data analytics, International Institute for Population Sciences(IIPS), Mumbai, India; ^2^ Department of Population and Development, International Institute for Population Sciences(IIPS), Mumbai, India

**Keywords:** body mass index, BMI-WC, abdominal obesity, risk categories, Indians

## Abstract

**Objective:** To determine the prevalence and predictors of combined BMI-WC disease risk categories among Indian adults.

**Methods:** The study utilizes data from Longitudinal Ageing Study in India (LASI Wave 1) with an eligible sample of 66, 859 individuals. Bivariate analysis was done to get the proportion of individuals in different BMI-WC risk categories. Multinomial logistic regression was used to identify the predictors of BMI-WC risk categories.

**Results:** Poor self-rated health, female sex, urban place of residence, higher educational status, increasing MPCE quintile, and cardio-vascular disease increased with increasing BMI-WC disease risk level while increasing age, tobacco consumption, and engagement in physical activities was negatively associated with BMI-WC disease risk.

**Conclusion:** Elderly persons in India have a considerable higher prevalence of BMI-WC disease risk categories which make them vulnerable to developing several disease. Findings emphasize the need of using combined BMI categories and waist circumference to assess the prevalence of obesity and associated disease risk. Finally, we recommend that intervention programs with an emphasis on urbanites wealthy women and those with a higher BMI-WC risk categories be implemented.

## Introduction

There has been a lot of discussion on the rising trends in generalized obesity, defined using body mass index (BMI) values (1, 2). At the same time, abdominal obesity, often used to define different obesity categories (3) is assessed using Waist Circumference (WC) and has been linked with biochemical risk markers, morbidity, and all-cause mortality across BMI categories (4). Obesity has been abundantly established as one of the risk factors for metabolic diseases such as hypertension, diabetes mellitus, insulin resistance, dyslipidemia, disability, and increased mortality among older adults (5–7). Obesity and its related complications are also detrimental to economic growth and development (8, 9). Estimates suggest that nearly 17 million people face premature mortality from non-communicable diseases related to preventable risk factors such as overweight/obesity, energy dense diets, lack of physical activity, and over-consumption of alcohol and tobacco (10). The escalating prevalence of overweight and obesity in developing countries like India has been occurring simultaneously with demographic and epidemiological transitions, wherein fertility and mortality are declining and lifestyle-related diseases are on the rise (11–13).

It is noteworthy that the rate of increase in overweight and obesity prevalence in India is higher than the global rate of increase (14). For example, between 1998 and 2020, the overweight population increased from 8.4% to 15.5% among women, and the prevalence of obesity increased from 2.2% to 5.1% over the same period (15–18). This fleet-footed increase has taken place concomitantly with considerable rises in the burden of non-communicable diseases (NCDs). Older adults with abdominal obesity have been reported to be at increased risk of cardiovascular diseases than their non-obese counterparts, independent of body mass index categories (19). Despite the widespread usage of body mass index as an indicator of overweight and obesity, recent studies have shown that abdominal obesity is a better measure of excess body fat than BMI (20, 21), especially among older adults because of age-dependent height decrease (22).

Studies suggest a combination of BMI and WC to predict the obesity-related disease than using only any one indicator (23). The remarkable rise in the population with increased BMI and waist circumference poses a significant health problem in the country. Moreover, research has shown that people with different BMI and high WC have a higher risk of developing a disease such as hypertension (24), and stroke (25). BMI and waist circumference can identify the highest risk phenotype of obesity than BMI and WC alone (26). The increased prevalence of overweight and obesity among older adults necessitates the assessment of related health issues to develop preventive and curative health strategies (27).

Different nutrition programs in India give more attention to undernutrition irrespective of the increase in the prevalence of overweight and obesity in the country. There is a lack of sufficient information regarding the prevalence and contributing factors of combined BMI and WC among adults in India. Thus, this study determines the prevalence of combined BMI-WC disease risk categories and associated socio-demographic factors among adult population in India. The information can be used as baseline evidence for program planners, policymakers, researchers, and organizations who are working on the prevention of chronic NCDs.

## Methods

### Data Source

Data in this study originated from the first wave of a prospective cohort ageing study “Longitudinal Ageing Study in India” (2017–18) (28). This biennial panel survey conducted in all Indian states and Union territories (UTs) adopts a multi-stage stratified area probability cluster sampling design with older adults age 45 and above and their spouses irrespective of their age as the eligible sample. This survey provides credible and comprehensive scientific evidence on economic status, chronic and symptom-based health conditions, functional and mental health, health insurance and healthcare utilization, family and social networks, employment and retirement, income and consumption, satisfaction, and life expectations. More details on the information provided by the survey can be found in report (28). For this study, we first merged individual files and biomarker files using one-to-one matching to assess the information on measured blood pressure and anthropometry measures such as height, weight, nutritional status, waist-to-hip ratio, and waist-hip circumference. The cases (*n* = 6,537) for which information was not available in the biomarker file were excluded from the analytical sample. After exclusion, the eligible sample size for the study was restricted to 66, 859 individuals aged 45 and above and their spouses irrespective of their age.

### Variables

The dependent variable used in this study is four combined BMI-WC categories constructed using BMI and WC cut-off points (29).

Waist circumference is often used as a surrogate marker of abdominal fat mass (20). Waist and hip circumferences were measured in centimeters using a Gulick tape according to standard protocols (28). Men and women were categorized as having abdominal obesity if they had a waist circumference of >102 cm and >88 cm, respectively. WHO considers men and women to be at an increased risk of metabolic complications if having a waist circumference of >102 cm and 88 cm (30). Further, the individuals were categorized into different risk categories based on abdominal obesity and Body Mass Index (BMI). The individual was categorized as follows: 1) “low risk” if normal BMI and did not have abdominal obesity 2) “increased risk” if overweight individuals without abdominal obesity 3) “high risk” if normal BMI and abdominally obese 4) “very high risk” if obese.

Socio-demographic characteristics include age, sex, marital status, place of residence, wealth index, caste, religion, region, Education, living arrangements, smoking, alcohol consumption and respondent’s physical activity. We classified age into four groups: <60 years, 60–69 years, 70–79 years and 80 and above. Individuals were categorized as male and female based on their sex. We categorized marital status as “currently married/living together” and “widowed/divorced/separated/never married.” The place of residence was taken as “rural’ and “urban.” The monthly *per capita* consumption expenditure was calculated based on the household expenditures on food and non-food items with the reference period of 30 and 365 days, respectively. The expenditures were further standardized to the 30-day reference period and were sorted as poorest, poorer, middle, richer and richest. Caste was grouped as Scheduled Castes (SC), Scheduled Tribes (ST), Other Backward Castes (OBC) and Others and religion was categorized as Hindu, Muslim, Christian and others based on their responses. The country was divided into six regions, i.e., North, Central, East, North-east, West and South based on the geographical location of the states. Concerning the educational status of individuals, we coded them as “no formal schooling,” “1–5 years of schooling,” “and 6–9 years of schooling” and “10 and above years of schooling.” Living arrangement was grouped at three levels: living alone, living with a spouse and living with a person other than the spouse. Self-rated health was evaluated from the question: Overall, how is your health in general? Would you say it is very good, good, fair, poor or very poor? The answer: very good or good was assigned with “good” while fair, poor or very poor was recoded as “poor.” The respondents were asked if they ever consumed alcohol or smoked. Alcohol consumption was categorized as lifetime abstainer, non-heavy drinker (consuming alcohol less than once a month or those who consume alcohol 1–3 days per month, 1–4 days per week, or 5 or more days per week but did not consume more than 5 standard drinks on any occasion in the past 30 days) and heavy drinker (those who consume at least 60 g or more (approximately 5 drinks) of pure alcohol on at least one occasion in the past 30 days). For smoking, their responses were recorded as yes or no. Respondents were asked about their involvement in moderate and vigorous physical activity. Those who reported being engaged in any vigorous and moderate physical activity for everyday were counted as being physically active. Respondents were recoded as not being physically active if they were not involved in any physical activity or those who were active more than once a week/once a week/one to three times a month.

Regarding the chronic disease status, this study utilizes information on self-reported ever diagnosed conditions/diseases by health professional such as Bachelors of Medicine and Bachelors of Surgery (MBBS), Doctor of Medicine (MD), Bachelors of Dental Surgery **(**BDS) and (Ayurveda, Yoga, Naturopathy, Unani, Siddha, and Homeopathy) AYUSH Only. The respondents were asked, “Has any health professional ever diagnosed/told you that you have the following disease conditions or disease”? The chronic conditions included in this study are diabetes, hypertension, heart disease, stroke, and high cholesterol. The respondent was considered to have a history of cardio-vascular disease if he had atleast one of the above-mentioned diagnosed chronic conditions. Blood pressure was measured using an Omron HEM 7121 BP monitor, adopting internationally comparable protocols. High blood pressure often termed Hypertension was assessed using the recommendations provided by WHO. The WHO classification system for blood pressure is: Normal: systolic <120 mmHg and diastolic <80 mmHg; pre-hypertension: systolic 120–139 mmHg and/or diastolic 80–89 mmHg and hypertension or high blood pressure: systolic ≥140 mmHg and/or diastolic/≥90 mmHg. Body mass index is calculated by dividing an individuals’ weight (in kilograms) by the square of their height (in meters). Height was measured in centimeters using a stadiometer, and weight was measured in kilograms using a Seca 803 digital weighing scale. Individuals were categorized as underweight (if BMI <18.5), normal (BMI 18.5–24.9), overweight (BMI 25–29.9) and obese if BMI was 30 and above.

### Statistical Analysis

Descriptive statistics were used to summarize the socio-demographic variables of the study participants using frequency and percentages. The given frequency is unweighted while the percentage distribution is weighted. Bivariate analysis was done to get the proportion of individuals in different BMI-WC risk categories. Finally, an analysis of the determinants of BMI-WC risk categories was carried out using multinomial logistic regression. Ideally, multinomial logistic regression is applied in a situation where the dependent variable is categorized into three and more nominal responses (31). Odds ratios (ORs) with 95% confidence intervals (CIs) were calculated for the dependent variables. All the statistical analyses were carried out using STATA version 17.

## Results

The variable distribution of the dependent and independent variables used in this paper has been demonstrated in Table 1. Out of the total eligible population, around 14% were alcohol users and a little more than one-third consumed tobacco. More than three-fifths rated their health as poor and fifty-eight percent of the participants were female. Three-fourths of the participants were currently married and a little more than half of the eligible participants were aged less than 60 years. Around two third of the eligible sample resided in rural areas and the distribution of participants in different wealth quintiles was almost the same. The highest proportion of respondents came from the Eastern region, followed by the Southern and central states with the lowest number of participants from the north-east. Around fifty percent of the respondents did not have any formal education and a majority of them resided with a spouse or other than the spouse. Around one-third of the participants had cardiovascular disease and 39% and 31% had pre-hypertension and hypertension respectively. We found that majority of the participants were physically active (64%).

**TABLE 1:** Distribution of study population by selected background characteristics, India, LASI-wave-1, 2017–18.

Sample characteristics		Frequency	Percentage
Age (in years)	<60 years	37,924	54.21
	60–69	17,626	27.3
	70–79	8,383	13.66
	80+	2,926	4.83
Sex	Male	28,220	41.86
	Female	38,639	58.14
Marital status	Currently married	51,874	76.44
	Widow/div/single	14,983	23.56
Years of schooling	No schooling	30,807	49.71
	1–5	12,292	17.66
	6–9	11,117	14.75
	10+	12,643	17.87
Place of residence	Rural	43,690	69.5
	Urban	23,169	30.5
MPCE quintile	Poorest	13,191	20.93
	Poorer	13,487	21.26
	Middle	13,515	20.42
	Richer	13,564	19.74
	Richest	13,102	17.65
Caste	SC	11,103	19.4
	ST	11,963	8.53
	OBC	25,378	45.67
	Others	18,415	26.4
Religion	Hindu	48,807	82.42
	Muslim	7,902	11.22
	Christian	6,761	2.9
	Others	3,389	3.45
Region	North	11,916	12.35
	Central	8,767	20.47
	East	11,926	23.98
	North-east	9,847	3.67
	West	8,673	15.76
	South	15,730	23.77
Living arrangement	Living alone	2,141	3.4
	Living with spouse	50,646	75.02
	Living with others	14,072	21.57
Blood pressure	Normal	18,798	30.31
	Pre-hypertension	26,096	39.02
	Hypertension	21,831	30.68
Physical activity	No	25,216	35.39
	Yes	41,548	64.61
Alcohol use	Life-time abstainer	55,716	86.03
	Non-heavy drinker	8,641	11.12
	Heavy drinker	2,425	2.85
Tobacco use	Never smoked	43,683	64.46
	Ever smoked	23,088	35.54
Cardiovascular Disease	No	43,786	67.41
	Yes	23,046	32.59
Self-rated health	Good	28,764	38.66
	Poor	38,063	61.34
	Total	66,859	

The prevalence of different BMI-WC disease risk categories is shown in Table 2. Overall, 44% of the sampled individuals and their spouses (irrespective of their age) were in high risk categories of combined BMI-WC. A higher percentage of alcohol consumers were at increased risk categories than no-alcohol users. Similarly, a higher proportion of non-tobacco users were in the “high risk” categories than tobacco users. For instance, 12% of non-tobacco users were at “very high risk” whereas only 4% of tobacco users were at “very high risk” disease. Moreover, the percentage of individuals who reported their health as “poor” were more in the high risk (26%) and very high risk (10%) categories than individuals reporting “good” health (22% and 9%, respectively). Percentage of females in high-risk and very high-risk categories of having metabolic disorders were five folds and three folds respectively to males. The percentage of respondents grouped by marital status and physical activity did not vary much for different disease risk categories. The population in the “very high risk” category was dominated by respondents aged <60 years. The proportion of urban individuals was higher than rural residents in the “increased risk, high risk and very high risk” category. As we moved from poorest to richest MPCE quintile, the percentage of people in high risk categories increased. For instance, around six percent of individuals from the poorest quintile were in the very high risk category whereas around 14% of the richest MPCE quintile respondents were in the very high risk category. The highest percentage of respondents from “Others” caste and Muslim and other religions were in high BMI-WC. Individuals having the cardiovascular disease were more in risk categories.

**TABLE 2:** Association between socio-demographic characteristics and combined body mass index and waist circumference (BMI-WC) disease risk categories, India, LASI-wave-1, 2017–18.

Background characteristics		BMI-WC risk category			
		Low risk	Increased risk	High risk	Very high risk
Age (in years)	<60 years	53.99	10.98	24.38	10.65
	60–69	57.58	8.32	25.87	8.23
	70–79	63.96	6.93	22.26	6.85
	80+	64.34	6.73	24.58	4.36
Sex	Male	71.86	15.82	7.87	4.46
	Female	45.93	5.37	36.01	12.68
Marital status	Currently married	57.17	10.71	22.74	9.38
	Widow/div/single	54.09	5.61	31.18	9.13
Years of schooling	No schooling	62.32	6.21	25.3	6.16
	1–5	58.55	9.4	23.68	8.37
	6–9	52.58	12.02	24.56	10.83
	10+	45.18	15.44	23.52	15.86
Place of residence	Rural	65.09	8.32	21.12	5.47
	Urban	40.42	12.11	30.92	16.56
MPCE quintile	Poorest	65.62	8.36	19.62	6.41
	Poorer	60.52	8.97	22.28	8.23
	Middle	58.09	8.77	25.63	7.51
	Richer	53.16	9.47	27.36	10.02
	Richest	45.48	12.61	27.48	14.42
Caste	SC	63.81	8.11	21.75	6.33
	ST	72.07	6.78	17.71	3.44
	OBC	55.65	10.47	23.87	10.01
	Others	49.31	9.96	29.1	11.63
Religion	Hindu	57.86	9.77	23.5	8.87
	Muslim	49.85	8.49	29.8	11.87
	Christian	57.07	9.14	26.11	7.68
	Others	47.28	10.65	29.35	12.72
Region	North	49.82	8.63	30.03	11.52
	Central	65.18	6.76	22.4	5.66
	East	65.2	8.14	21.24	5.42
	North-east	69.42	9.69	17.39	3.5
	West	52.73	9.96	26.81	10.5
	South	46.82	13.27	25.65	14.27
Living arrangement	Living alone	59.14	6.5	27.54	6.82
	Living with spouse	57.15	10.77	22.69	9.39
	Living with others	53.59	5.63	31.3	9.48
Blood pressure	Normal	65.67	6.73	21.09	6.52
	Pre-hypertension	56.47	10.08	24.38	9.07
	Hypertension	49.1	11.47	27.5	11.91
Physical activity	No	57.93	9.49	23.63	8.95
	Yes	55.78	9.71	24.99	9.52
Alcohol use	Life-time abstainer	53.82	9.16	26.83	10.18
	Non-heavy drinker	73.61	13.36	9.27	3.76
	Heavy drinker	80.35	10.57	7.05	2.03
Tobacco use	Never smoked	49.36	9.52	29.37	11.74
	Ever smoked	71.84	9.88	14.16	4.12
Cardiovascular Disease	No	63.98	9.19	20.27	6.57
	Yes	43.19	10.45	32.1	14.26
Self-rated health	Good	57.2	11.5	22.34	8.96
	Poor	56.06	8.38	25.99	9.57
	Total	56.51	9.64	24.53	9.32

Table 3 shows the associated risk factors with different abdominal risk category among elderly people, with odd ratios and corresponding 95% confidence interval. Having poor self-rated health, female sex, urban place of residence, higher educational status, increasing MPCE quintile, and presence of cardio-vascular disease increased the risk of being in higher risk levels of BMI-WC while increasing age, tobacco consumption, engagement in physical activities was negatively associated with BMI-WC disease risk. Living with spouse or living with anyone else than the spouse increased the risk of being in higher BMI-WC categories than individuals living alone. Pre-hypertensive respondents were 1.56 and 1.78 times, respectively more likely to have BMI-WC “high“ and “very high risk“ categories in reference to individuals with normal blood pressure. At the same time, hypertensive respondents were 1.98 and 2.53 times, respectively more likely to have BMI-WC “high“ and “very high risk“ categories in comparison to individuals with normal blood pressure.

**TABLE 3:** Adjusted odds ratios (OR) for the association between socio-demographic characteristics and combined body mass index and waist circumference (BMI-WC) disease risk categories: results from multinomial regression, India, LASI-wave-1, 2017–18.

Background characteristics		Low risk vs. Increased risk	Low risk vs. High risk	Low risk vs. Very high risk
Age (in years)	<60 years^®^			
	60–69	0.68*** (0.63, 0.73)	0.99 (0.94, 1.05)	0.72*** (0.66, 0.78)
	70–79	0.48*** (0.43, 0.53)	0.82*** (0.76, 0.89)	0.43*** (0.37, 0.48)
	80+	0.34*** (0.27, 0.42)	0.73*** (0.63, 0.84)	0.33*** (0.26, 0.41)
Sex	Male^®^			
	Female	0.6*** (0.55, 0.64)	11.18*** (10.4, 12.01)	5.98*** (5.45, 6.57)
Marital status	Currently married^®^			
	Widow/div/single	0.97 (0.78, 1.2)	0.96 (0.81, 1.14)	1.03 (0.81, 1.3)
Years of schooling	No schooling^®^			
	1–5	1.24*** (1.13, 1.36)	1.27*** (1.19, 1.35)	1.61*** (1.46, 1.77)
	6–9	1.51*** (1.39, 1.65)	1.54*** (1.43, 1.65)	2.25*** (2.04, 2.49)
	10+	1.78*** (1.63, 1.95)	1.94*** (1.8, 2.09)	2.73*** (2.47, 3.02)
Place of residence	Rural^®^			
	Urban	1.67*** (1.57, 1.78)	1.71*** (1.63, 1.8)	2.65*** (2.46, 2.84)
MPCE quintile	Poorest^®^			
	Poorer	1.14** (1.03, 1.26)	1.18*** (1.09, 1.27)	1.21*** (1.07, 1.36)
	Middle	1.12** (1.01, 1.24)	1.28*** (1.19, 1.38)	1.36*** (1.21, 1.52)
	Richer	1.33*** (1.2, 1.47)	1.46*** (1.35, 1.57)	1.78*** (1.59, 2)
	Richest	1.62*** (1.46, 1.79)	1.72*** (1.59, 1.86)	2.21*** (1.97, 2.48)
Caste	SC^®^			
	ST	0.87** (0.77, 0.98)	0.72*** (0.66, 0.79)	0.76*** (0.65, 0.88)
	OBC	1.06 (0.97, 1.17)	1.06 (0.98, 1.13)	1.13** (1.02, 1.26)
	Others	1.13** (1.03, 1.25)	1.35*** (1.25, 1.46)	1.5*** (1.34, 1.67)
Religion	Hindu^®^			
	Muslim	1.03 (0.93, 1.13)	1.56*** (1.45, 1.67)	1.38*** (1.25, 1.53)
	Christian	1.01 (0.9, 1.14)	1.17*** (1.06, 1.29)	1.18** (1.03, 1.35)
	Others	1.57*** (1.38, 1.79)	1.49*** (1.34, 1.66)	1.87*** (1.63, 2.15)
Region	North^®^			
	Central	0.72*** (0.64, 0.82)	0.66*** (0.6, 0.72)	0.48*** (0.42, 0.55)
	East	0.8*** (0.71, 0.89)	0.57*** (0.53, 0.62)	0.4*** (0.36, 0.46)
	North-east	1.08 (0.96, 1.22)	0.44*** (0.4, 0.48)	0.29*** (0.25, 0.34)
	West	1.06 (0.95, 1.18)	0.8*** (0.74, 0.87)	0.78*** (0.7, 0.87)
	South	1.57*** (1.42, 1.72)	0.86*** (0.8, 0.92)	0.89** (0.81, 0.99)
Living arrangement	Living alone^®^			
	Living with spouse	1.42** (1.09, 1.86)	1.39*** (1.13, 1.7)	1.73*** (1.29, 2.33)
	Living with others	1.26** (1.02, 1.56)	1.37*** (1.19, 1.56)	1.52*** (1.22, 1.88)
Blood pressure	Normal^®^			
	Pre-hypertension	1.53*** (1.41, 1.66)	1.56*** (1.47, 1.65)	1.78*** (1.63, 1.94)
	Hypertension	1.91*** (1.76, 2.08)	1.98*** (1.86, 2.11)	2.53*** (2.31, 2.77)
Physical activity	No^®^			
	Yes	0.95 (0.89, 1.01)	0.88*** (0.84, 0.93)	0.82*** (0.76, 0.88)
Alcohol use	Life-time abstainer^®^			
	Non-heavy drinker	0.9** (0.83, 0.99)	1.03 (0.93, 1.14)	1.04 (0.91, 1.2)
	Heavy drinker	0.76*** (0.65, 0.88)	0.88 (0.73 1.05)	0.75** (0.58, 0.99)
Tobacco use	Never smoked^®^			
	Ever smoked	0.77*** (0.72, 0.83)	0.9*** (0.85, 0.96)	0.67*** (0.61, 0.74)
Cardiovascular Disease	No^®^			
	Yes	1.53*** (1.43, 1.63)	1.86*** (1.77, 1.95)	2.52*** (2.36, 2.71)
Self-rated health	Good^®^			
	Poor	0.9*** (0.84, 0.95)	1.06** (1.01, 1.11)	1.1*** (1.03, 1.18)
	cons_	0.07 (0.05, 0.09)	0.02 (0.02, 0.03)	0.01 (0, 0.01)

***Significant 99% confidence level, ** significant at 95% confidence level, * significant at 90% confidence level, ^®^ Reference Category.

## Discussion

The present study deals with the prevalence and correlates of combined BMI-WC categories, which is one of the most important risk factors for a host of non-communicable diseases such as diabetes mellitus, hypertension, cancer and cardiovascular diseases. Additionally, health problems associated with NCDs have considerable negative ramifications for economic growth and development highlighting their importance to be studied. Thus, in view of facilitating the reduction of threats related to this issue, especially in developing countries, it is pertinent to identify the factors that increase BMI-WC disease risk level.

Findings from our study show that while nearly half of the older adults aged less than 60 years were in the low combined BMI-WC risk category, 10% at increased risk, 25% at high risk and 9 percent older adults were at very high risk. Our findings showed that individuals consuming alcohol and tobacco were at lower elevated BMI-WC risk than their non-consuming counterparts. Both abdominal obesity and general obesity have been reported to be highly correlated with alcohol consumption (32). A plethora of cross-sectional studies have evaluated the role played by alcohol consumption in the development of abdominal obesity, but the results have been inconclusive (33, 34). Some studies have a found a statistically significant and positive relationship between the two (35, 36) whereas others have found negative to nil association between alcohol consumption and abdominal obesity in both men and women (37, 38).

Smoking and obesity are identified as important risk factors for several non-communicable diseases and even mortality at older ages. A negative association was observed between smoking habits and increasing BMI-WC risk level. However, mixed evidence exists on the association between smoking and obesity. A study on the prevalence of obesity among males suggests that nicotine consumption could lead to an increase in metabolism both during rest and mild physical activity (39). However, there is no evidence to suggest that nicotine enhances body energy expenditure in the long term. Another set of studies reports similar basal metabolic rates in smokers and non-smokers (40, 41). Similarly, Yun et al. also suggest that those who smoke weigh less than those who do not smoke and they tend to put on weight after they quit smoking (39).

Finding that individuals reporting “poor” self-rated health were at higher odds of being in increased BMI-WC risk levels is in line with past studies (42–44). Self-rated health is a crucial indicator of general health that is based on both experience of diseases and its consequences (43). Interestingly, our findings show that females were more likely to have high disease risk. Several previous studies have also reported that the distribution of body fat differs in male and female. Despite having lower body mass, female tend to have more body fat than male (44, 45). Our study reported that the odds of disease risk were lower for single individuals. Previous studies from different settings in Sudan, Tanzania, West Africa, Brazil, and China have also endorsed this finding (46–50). One major explanation behind this could be a change in eating habits after marriage. Decreasing odds of high combined BMI-WC risk categories with the older ages and increasing risk of combined BMI-WC were observed with higher educational attainment.

Urbanites were at higher risk of having intermediate and higher BMI-WC risk levels and the risk increased by increasing the BMI-WC risk levels. One possible explanation might be that urban residents have a sedentary lifestyle, less engagement in physical activities, and are mostly employed in less labour-intensive occupations, accompanied by the consumption of energy dense foods (51), as compared with their rural counterparts. Another probable explanation could be that urban area provides ease of access to food and escape from physical labour (52) coupled with access to technologies requiring less energy, together with the availability of calorie intensive and energy dense foods, and limited space for physical activities. In this study, persons from wealthier homes were at high and very high risk of having BMI-WC risk categories. This conclusion may be explained by the fact that wealthy individuals have better and more reliable access to food, less physical exercise, and a preference for “Western” diets. Contradictory results have been found on the relationship between different BMI-WC and wealth status. According to research from rural China and West Africa (49, 50), abdominal obesity is more common in the impoverished than in the wealthy. Findings that individuals with cardio vascular disease and high blood pressure were at higher risk of combined BMI-WC risk categories are not new (53). Zhu et al. reported higher ORs for CVD risk factors and other metabolic risk (54). The huge proportion of individuals in combined BMI-WC risk categories implies higher healthcare costs posing significant challenges in reducing the burden of healthcare costs for poor disadvantaged populations.

When evaluating the study’s findings, some restrictions should be addressed. First, memory bias may exist since participants self-reported their demographic, behavioural, and certain health factors. Second, the temporal link between combined BMI-WC risk levels and cardio vascular disease may not be well established due to the cross-sectional study design. Third, the survey does not attempt to assess participant food habits or medication usage, which may be crucial in determining BMI-WC risk levels and common cardiac metabolic risk factor. Despite these drawbacks, the current study provides information on the BMI-WC risk levels and highlight the importance of using combined BMI and WC risk categories to assess the obesity related disease risk in India. Moreover, the findings are of importance in formulating the need-based intervention programmes aimed at reducing the risk of cardiovascular disease with an emphasis on overweight and obese individuals.

### Conclusion

In conclusion, a considerable proportion of Indian are in high BMI-WC disease risk categories. Additionally, there is a strong correlation between elevated BMI-WC disease risk and cardio vascular disease risk. In order to assess the dietary causes of this rising burden of disease risk, more research is required. Findings also emphasize the need of using combined BMI categories and waist circumference to assess the prevalence of obesity and associated disease risk. Future research may reevaluate the association using a prospective longitudinal research strategy, in which participants are followed over time in order to record changes in the morbidity condition among these individuals with elevated BMI-WC risk levels. Finally, we recommend that intervention programs with an emphasis on urbanites wealthy women and those with a higher BMI-WC risk categories be implemented. The improvement of older people’s nutritional status and the promotion of good lifestyle behaviors, such as eating well and staying active, should also be part of public health interventions.
